# Clinicopathologic features of Good’s syndrome: Two cases and literature review

**DOI:** 10.1515/med-2021-0256

**Published:** 2021-04-01

**Authors:** Yi-Dan Chen, Zhong-Hui Wen, Bing Wei, Shu-Yuan Xiao, Yu-Fang Wang

**Affiliations:** Department of Rheumatology and Immunology, West China Hospital, Sichuan University, Chengdu, Sichuan, People’s Republic of China; Department of Gastroenterology, West China Hospital, Sichuan University, Chengdu, Sichuan, People’s Republic of China; Department of Pathology, West China Hospital, Sichuan University, Chengdu, Sichuan, People’s Republic of China; Department of Pathology, The University of Chicago, Chicago, IL, United States of America

**Keywords:** Good’s syndrome, diarrhea, thymoma, opportunistic infection, immunodeficiency disease, common variable immunodeficiency

## Abstract

**Background:**

Good’s syndrome (GS) is an immunodeficiency disease, causing thymoma, low or absent B-cells, hypogammaglobulinemia, and defects in cell-mediated immunity. The most common clinical presentation is recurrent infection, followed by refractory diarrhea, due to the immunodeficiency. However, there are only few reports on intestinal endoscopy and pathology.

**Case summary:**

We report here two typical GS cases with diarrhea as the prominent manifestation. Both cases presented with thymoma combined with immunodeficiency, characterized by hypogammaglobulinemia, low or absent B lymphocytes, and decreased T-cells with inverted CD4^+^/CD8^+^ T-cell ratio, while two GS patients were evaluated by endoscopy revealed mucosal edema and fine-granular or nodular appearance changes in the small intestine. Histological examination showed chronic inflammation and villous atrophy. A very interesting finding is that the inflammatory cell infiltration in the two GS cases was different. In one case, predominantly CD138^+^ plasma cells with only scattered CD3^+^ T-cells infiltration were revealed, while in another, it showed predominantly T-cells infiltration without plasma cells in the lamina propria. Although GS cases shared various clinical characteristics with common variable immunodeficiency (CVID) cases, they still differed from CVID cases in terms of its late onset, lack of familial clusters, low or absent peripheral blood B lymphocytes, absence of lymphoid hyperplasia, and plasma cells infiltration in the lamina propria in some patients. Although both patients had been diagnosed previously with recurrent diarrhea, respiratory infection, and thymoma, the association between these conditions and the possibility of GS was not recognized. The patients had remained misdiagnosed for 2 and 4 years, respectively, even after receiving the diagnosis of thymoma. The rarity of GS was likely the primary cause for the lack of disease recognition. Reporting of these cases will help to alert clinicians and raise awareness of this disease.

**Conclusion:**

GS should be considered among the differential diagnoses for patients with unexplained recurrent diarrhea and opportunistic infection. Although it was regarded as a subset of CVID with thymoma, GS had a different clinical-pathological feature from CVID.

## 1 Introduction

Good’s syndrome (GS), first described in 1955 by Dr Robert Good, denotes the coexistence of thymoma with immunodeficiency [1]. It was originally regarded as a subset of the common variable immunodeficiency disorders (CVIDs) but accompanied by thymoma; in 1999, it was classified by the expert committee of the World Health Organization/International Union of Immunological Societies as a distinct primary immunodeficiency disorder, separate from CVID [2].

GS remains a relatively rare immunodeficiency condition, underlying a lack of recognition. Publications of this disease are single case reports or small case series. The largest primary case series of GS was an investigation of 78 cases from the national UK-Primary Immune Deficiency (UKPID) registry from 2009 to 2018 [3]. There were also some literature reviews such as a review about 152 cases published before 2010 and a review of 47 Chinese patients before 2017 [4]. All of these surveys focused on the clinical and laboratory features of patients with GS, and there is no report describing and analyzing the endoscopic and pathological features of GS.

A search of our hospital’s database (from January 1, 2008, to December 31, 2018) found only two cases, both of which had been admitted because of recurrent diarrhea. Herein, we present these typical GS cases with refractory diarrhea.

## 2 Case report

### 2.1 Case 1

A 58-year-old Chinese woman was referred to our Gastroenterology Department for recurrent diarrhea. The patient reported suffering from recurrent diarrhea, about three to four times a day and without tenesmus and bloody stools, for the past 4 years. Treatment with antibiotics, probiotics, and traditional Chinese medicine had been ineffective. Two years ago, the patient had undergone chest computed tomography (CT) in a local hospital to investigate recurrent cough and intermittent dyspnea. The scan showed a mediastinal mass and pneumonia with bronchiectasis. Resection of the mass led to the diagnosis of type AB thymoma. Postoperatively, the patient’s diarrhea continued, but upper endoscopy and colonoscopy performed in a local hospital yielded negative findings.

Physical examination showed emaciation, with a weight loss of nearly 15 kg before her admission. Some moist rales were heard in both lower lung fields. There were no signs of abdominal tenderness, hepatosplenomegaly, or edema for either lower limb.

Blood tests on admission showed a significant decrease in serum electrolyte levels including hyponatremia, hypokalemia, hypomagnesemia, hypophosphatemia, and hypocalcemia. Low serum levels of albumin and globulin were also observed (Table 1). The stool routine showed negative findings for white blood cells, red blood cells, and myocytes. No definite pathogens were identified from stool bacteria cultivation. Grocott’s methenamine silver staining gave negative findings for fungal organisms. Meanwhile, the results from tests for human immunodeficiency virus (HIV), rubella virus, cytomegalovirus (CMV), herpes simplex virus (HSV), Epstein–Barr virus (EBV), *Aspergillus*, and *Cryptococcus* were all negative. Tests for antinuclear antibody and extractable nuclear antigen antibody spectrum produced negative results. However, further examination showed hypoimmunoglobulinemia with low serum levels of IgG, IgA, and IgM. The B cell lineage was profoundly deficient (Table 1). An inverted CD4^+^/CD8^+^ T-cell ratio was also observed.

**Table 1:** Clinical characteristics of the patients in two cases

	Case 1	Case 2	Reference range
Gender	Female	Male	N/A
Age (years)	58	47	N/A
Symptoms	Diarrhea, cough, dyspnea	Diarrhea, cough	N/A
Sites of infection	Lung	Lung	N/A
Treatment	IVIG + antibiotics	IVIG + antibiotics	N/A
Outcome	Remission	Remission	N/A
**Analyte**			
Absolute B-cell count	50	102	175–332 cells/µL
CD4^+^/CD8^+^ T-cell ratio	0.4	0.16	0.97–2.31
Sodium	120	136	135–150 mmol/L
Potassium	2.81	2.32	3.5–5.3 mmol/L
Magnesium	0.42	0.83	0.67–1.04 mmol/L
Phosphorus	0.38	0.36	0.81–1.45 mmol/L
Calcium	1.57	1.79	2.1–2.7 mmol/L
Albumin	31.4	36	40–55 g/L
Globulin	15.7	18	20–40 g/L
IgG	0.49	0.56	8–15.5 g/L
IgA	<66.7	1,460	836–2,900 g/L
IgM	<41.70	1,240	700–2,200 g/L

N/A = not available.

**Figure 1:**
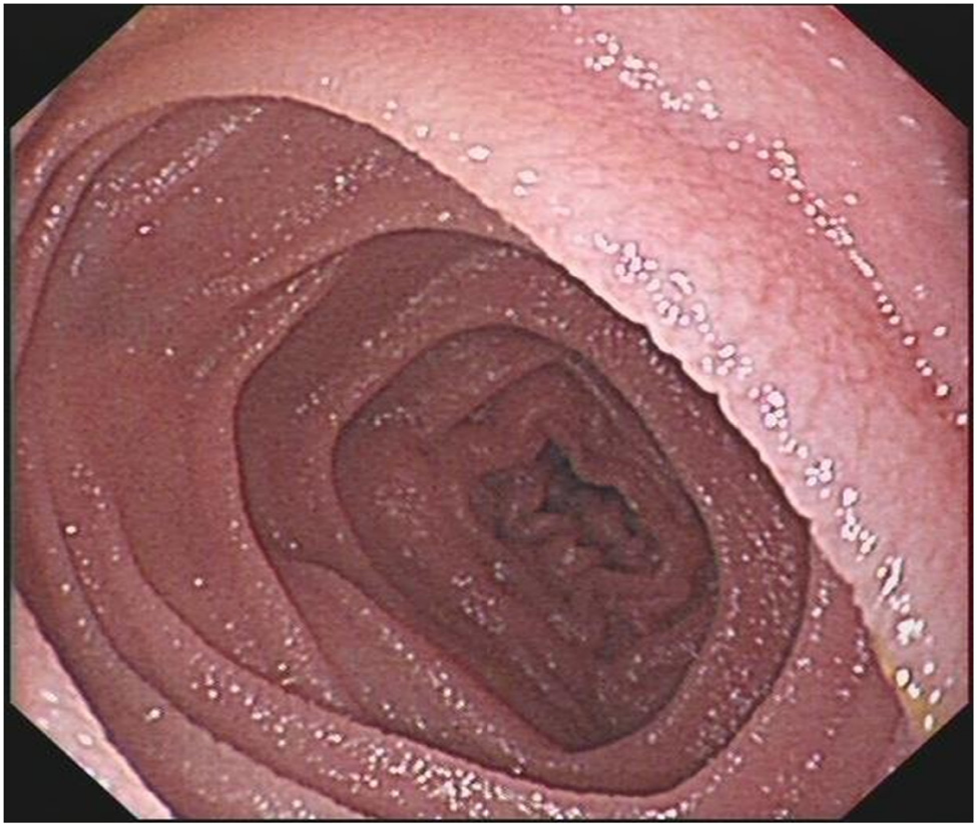
Mucosal edema and fine-grained appearance diffusely distributed in the small intestine.

**Figure 2:**
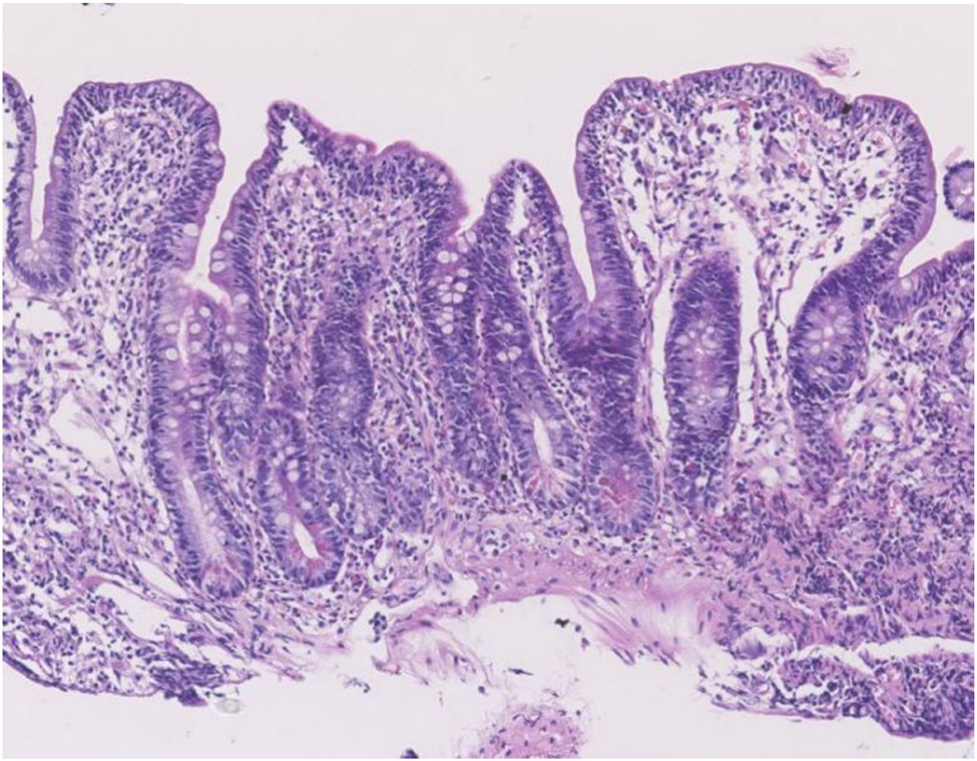
Histopathological findings in the jejunum biopsy: villous blunting and chronic inflammatory infiltrate in lamina propria (hematoxylin and eosin stain).

**Figure 3:**
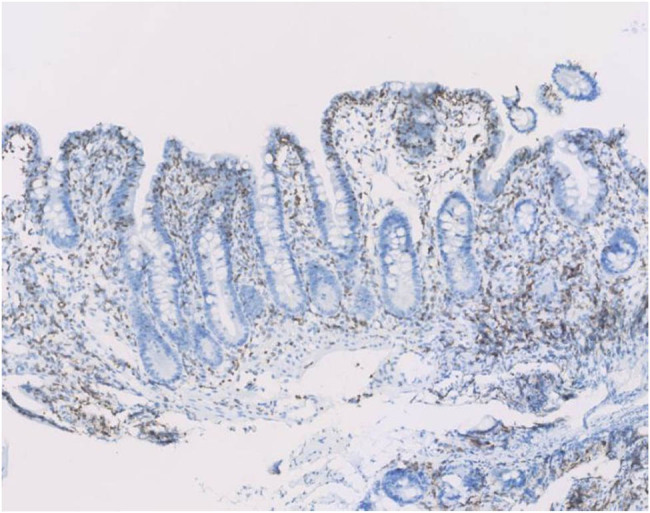
CD3 immunostaining shows predominant T-cells infiltration in the jejunum mucosa.

**Figure 4:**
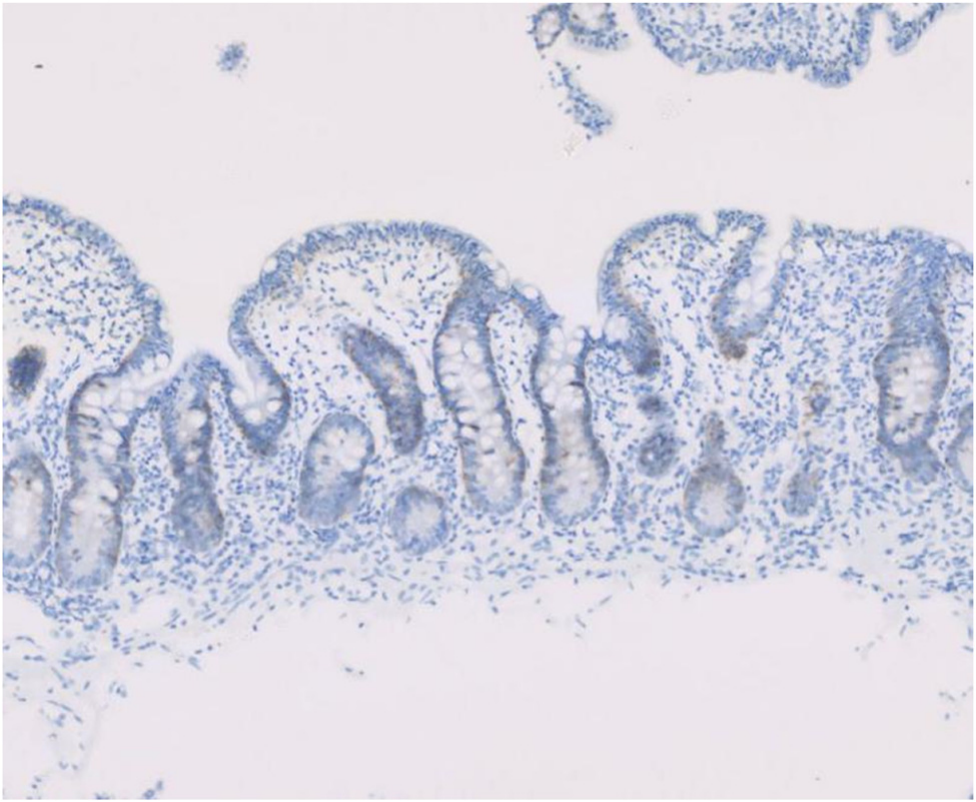
Lack of plasma cells in the lamina propria of the jejunum (CD138 immunohistochemistry).

Enteroscopic findings showed mucosal edema and fine-grained appearance diffusely distributed in the proximal small intestine (Figure 1). Histopathological examination of jejunum mucosal biopsies showed mucosal atrophy, villous blunting, focal dilation of the lymphatics, and mild-to-moderate chronic inflammatory cellular infiltration with the presence of CD3 cells and the absence of plasma cells (Figures 2–4).

Given the history of repeated respiratory infections with bronchiectasis, hypoimmunoglobulinemia, B lymphopenia, and thymoma, the diagnosis of GS was made. After treatment with antibiotics, fluid infusion, and delivery of balanced electrolytes, the patient improved greatly, with the relief of cough and diarrhea. The patient was then asked to start on regular administration of intravenous immunoglobulin (IVIG) treatment. Despite the patient’s consent to IVIG treatment, the time routine was irregular. During this period, the patient would develop cough, sputum, and diarrhea recurrence, which responded to anti-infective management and the IVIG. Overall, during the 1-year follow-up, the patient experienced a drastic improvement in her quality of life.

### 2.2 Case 2

A 46-year-old Chinese man was admitted with a 5-year history of recurrent diarrhea and weight loss. The patient reported suffering from diarrhea for about four to five times a day but without tenesmus, abdominal pain, or bloody stools. Four years before presentation, the patient had been diagnosed in the local hospital with “thymoma” following a complaint of chest pain. At that time, he underwent surgical resection of the thymoma; however, recurrent diarrhea became aggravated in the postoperative period and showed an unsatisfactory response to therapy. Importantly, the patient also became susceptible to respiratory infections.

Physical examination showed severe malnutrition and emaciation, with a body mass index of 16.8 kg/m^2^. Moist rales could be heard in both lungs. The patient’s abdomen was flat and soft, with active bowel sounds. There were no signs of abdominal tenderness or hepatosplenomegaly.

On admission, stool routine, bacterial culture, and parasite test of this patient showed negative results. Decreases in the serum electrolyte levels including potassium, phosphorus, and serum calcium were revealed with a decline in the serum levels of albumin and globulin. The serum level of IgA, IgM, and IgE was normal, whereas hypoimmunoglobulinemia was observed with low serum levels of IgG (Table 1). There was an inverted CD4^+^/CD8^+^ T-cell ratio. The results of the tests for HIV, HSV, *Toxoplasma gondii* antibody, CMV, EBV, rubella virus, *Aspergillus*, and *Cryptococcus* were negative. Tuberculin skin test also showed a negative result. Sputum cultures revealed *Haemophilus influenzae* and *Escherichia coli*. Chest CT scan showed the bilateral lower bronchus to be slightly dilated with infection. Abdominal CT showed that part of the gastrointestinal wall was swollen, and the middle end of the transverse colon was locally thickened.

**Figure 5:**
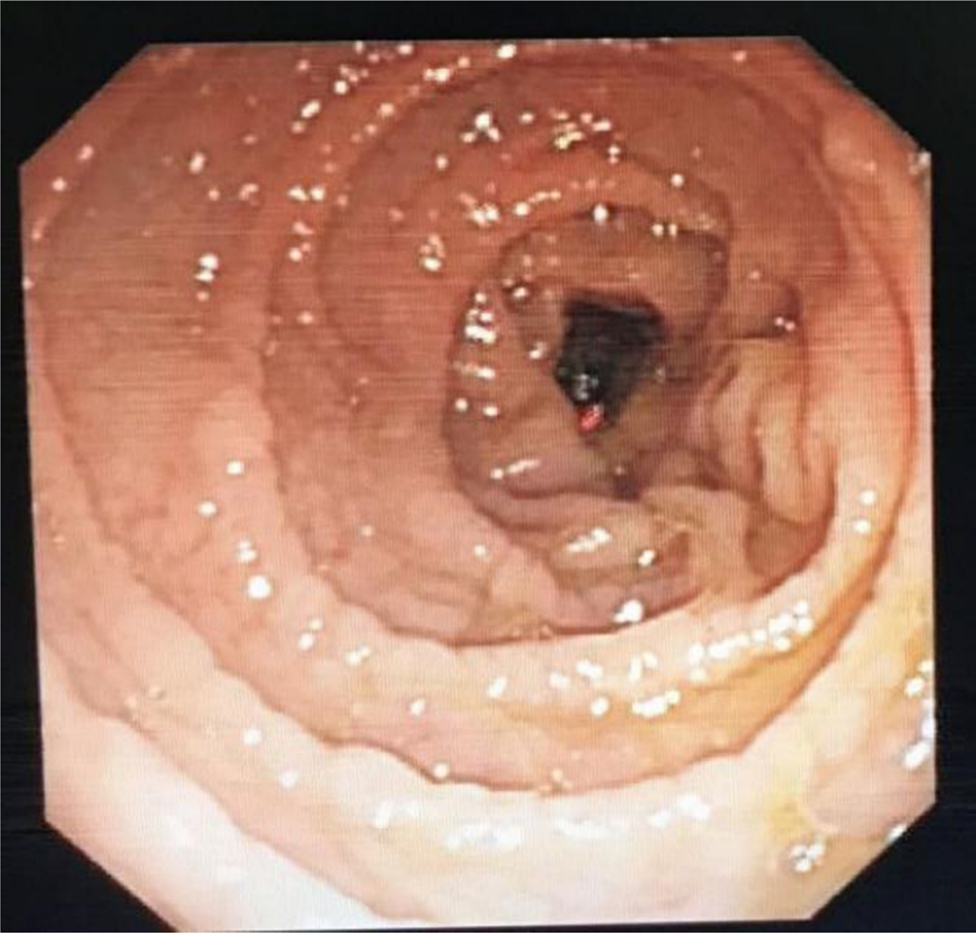
Nodular changes in the descending portion of the duodenum.

**Figure 6:**
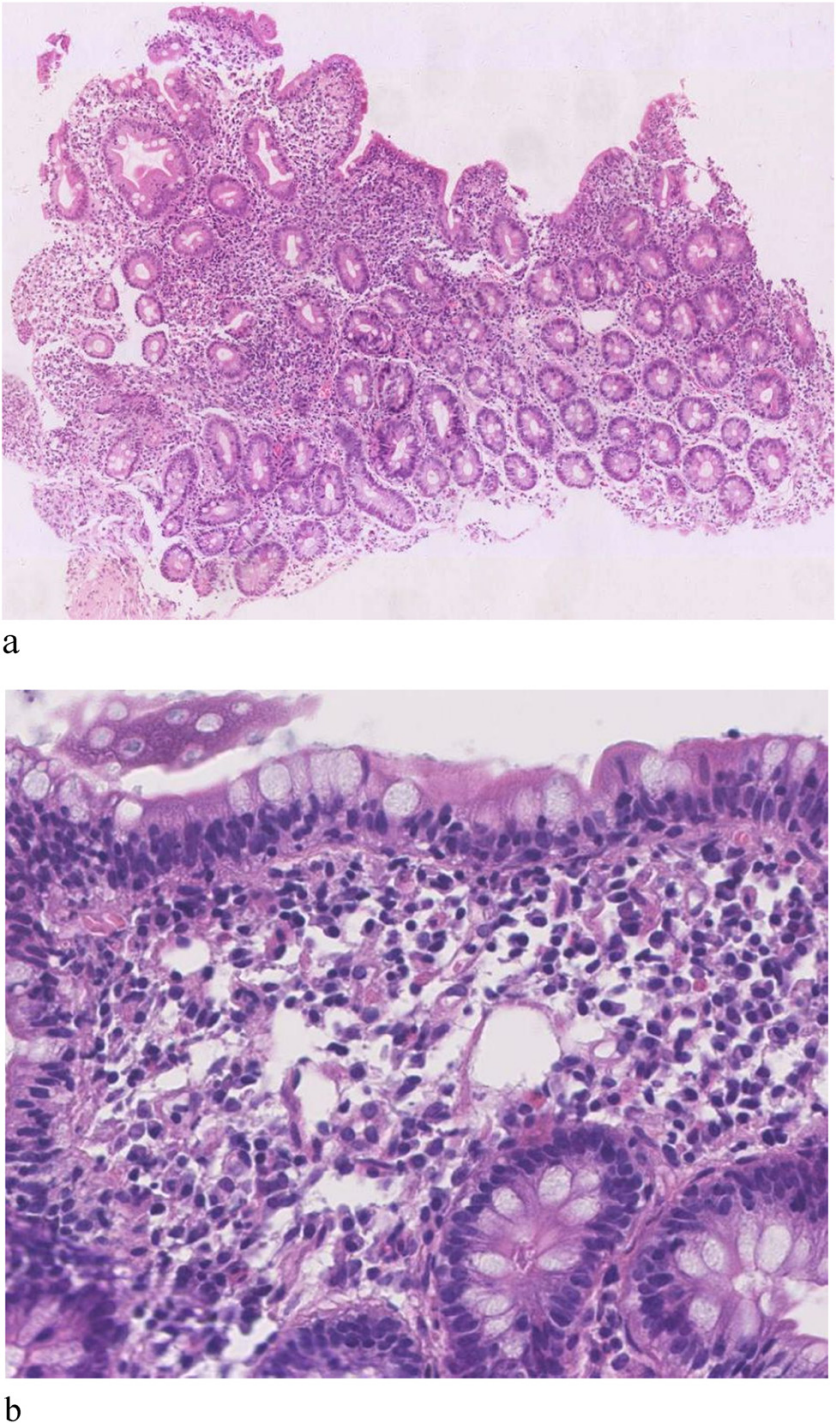
Histopathological findings in mucosal biopsies: (a) chronic inflammation of descending duodenum and villous blunting. (b) Biopsy from terminal ileum showing infiltration of plasma cells in the lamina propria (hematoxylin and eosin stain).

**Figure 7:**
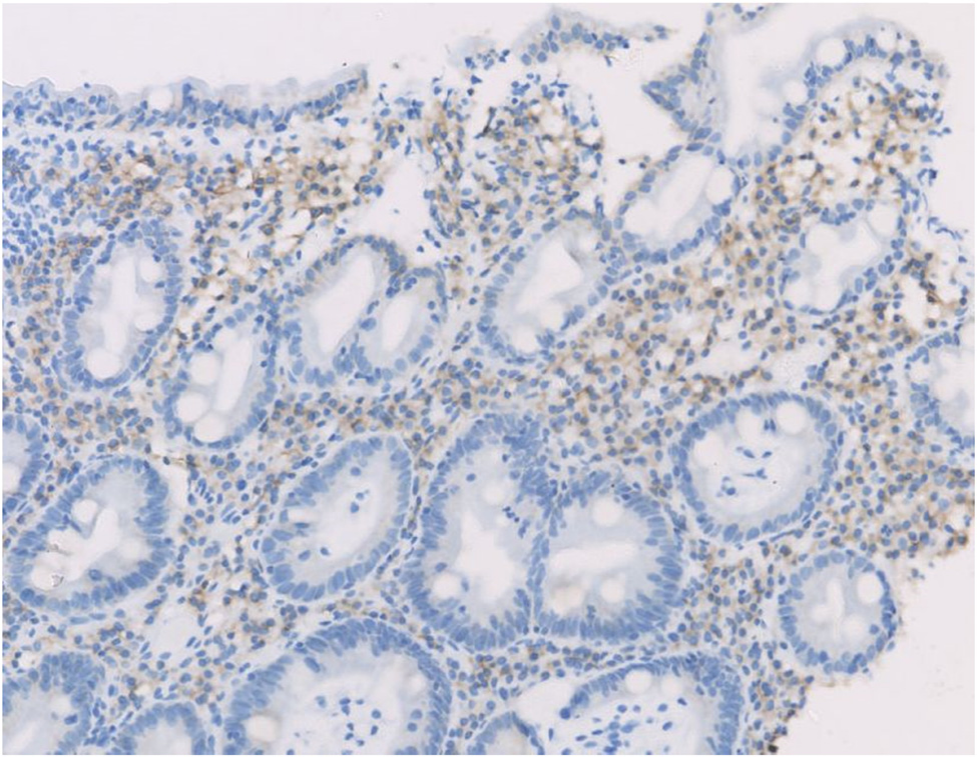
Plasma cells infiltrating in the mucosa are positive immunohistochemical staining for CD138.

**Figure 8:**
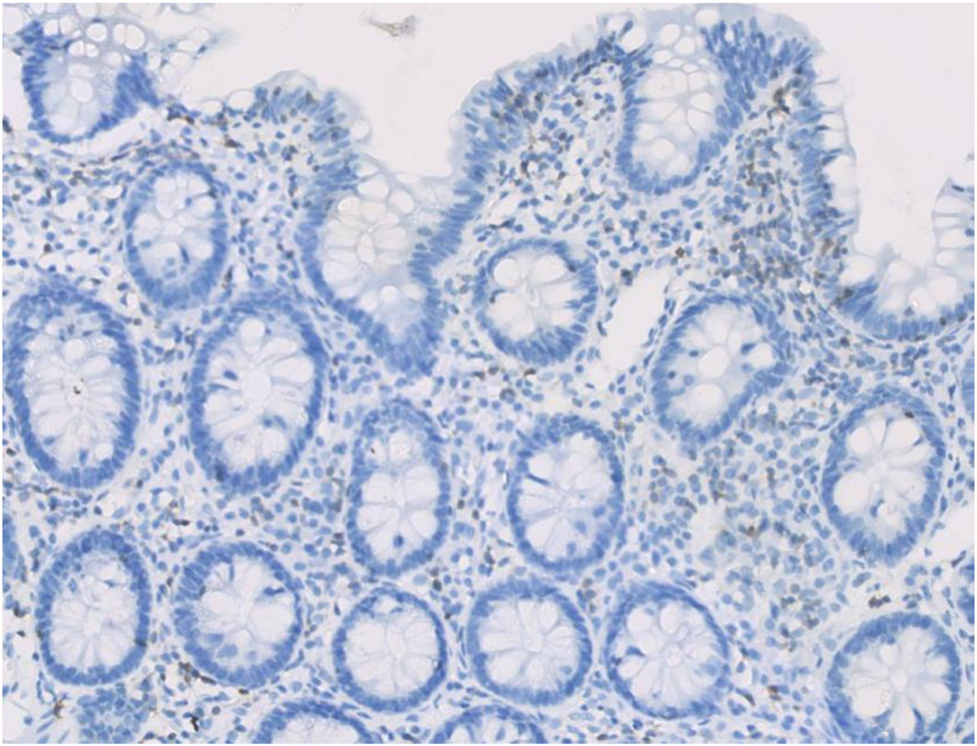
Some scattered CD3^+^ T-cells infiltration in the lamina propria of mucosa.

Gastroscopy revealed nodular changes in the descending duodenum (Figure 5). Colonoscopy presented focal hemorrhagic plaques in the transverse colon, with normal terminal ileum. Capsule endoscopy revealed extensively swollen and chapped mucosa, extending from the duodenum to the jejunum. Histopathological examination of the mucosa showed villous blunting in the descending duodenum and moderate chronic inflammation in the lamina propria of both descending duodenum and terminal ileum (Figure 6a and b). The inflammatory infiltrate contains CD138^+^ plasma cells, some scattered CD3^+^ T-cells, and eosinophils (Figures 7 and 8).

Intravenous nutritional support, antibacterial therapy, and correction of electrolyte disturbance were administered. The diarrhea remained despite the improvement in patient’s cough and expectoration. The patient’s medical history of thymoma prompted consideration of whether the diarrhea was associated with immunodeficiency. Hence, the diagnosis of GS was considered. Ultimately, the patient’s diarrhea improved after regular administration of monthly IVIG treatment.

The research related to human use has been complied with all the relevant national regulations, institutional policies, and in accordance the tenets of the Helsinki Declaration, and it has been approved by the authors’ institutional review board or equivalent committee.

Written informed consents were obtained from both of the patients for the publication of the data.

## 3 Discussion

One-third of patients with thymoma have no symptoms. The second third of patients have symptoms caused by the compression of surrounding organs by an expansive mass. The last third, and possibly up to one-half, of patients may have autoimmune and hematological abnormalities such as myasthenia gravis (estimated to affect 30–45%), thymoma-associated multiorgan autoimmunity, pure red cell aplasia, and GS [5,6].

GS has been defined as thymoma combined with immunodeficiency, characterized by hypogammaglobulinemia, low or absent B lymphocytes, decreased T-cells, inverted CD4^+^/CD8^+^ T-cell ratio, and reduced T-cell mitogen proliferative responses [7,8]. The association of thymoma and immunodeficiency has not been well explained, and its underlying mechanism remains largely unknown [9]. The immunological findings in GS patients have generally involved humoral immunity, such as few or absent B-cells and hypogammaglobulinemia. Low or absent peripheral blood B lymphocytes were reported in 87% of GS patients in one study [10], as opposed to the mature B-cell count having been often normal in patients with CVID. Both of our patients had decreased B-cell count. Most patients, including the first patient in our report, have reduced serum IgG, IgA, and IgM, while the cases reported by Watts and Kelly [11] included some patients who had normal IgA levels. The second patient in our study also had only reduced serum IgG level, with normal IgA and IgM levels. Immunological defects in GS patients also affected cellular immunity, such as decreased T-cells, inverted CD4^+^/CD8^+^ T-cell ratio, and reduced T-cell mitogen proliferative responses. An inverted CD4^+^/CD8^+^ T-cell ratio appears to be very common in both GS patients and patients with CVID, and it was also observed in our two patients.

Patients with GS usually present in the fourth or fifth decade of life and cases in childhood are rare [12]. Most reports [11,12] indicate no significant differences between the incidence in males and females. In contrast, however, a systematic review from China that assessed 47 patients from 27 studies found that Chinese females were more inclined to suffer GS than their male counterparts. It is also reported that the most common histologic type of thymoma in GS is type AB (50%), followed by type A (36%), type B2 (7%), type B3 (4%), and malignant thymoma (4%). Both of our cases had histologic type AB. The clinical manifestations of GS are similar to those seen in patients with CVID, cases of which also experience recurrent infections or diarrhea. However, compared to CVID, GS cases have a higher frequency of invasive bacterial infections and autoimmune complications.

Infections are the main characteristic of GS, and they can include bacterial, viral (i.e., CMV, EBV), and fungal infections. Watts and Kelly [11] reviewed 51 patients with GS and reported that *H. influenzae* (24%) was the most frequently isolated pathogen from patients with bronchiectasis. Viral infections were found in 40% of the GS patients, and the most common pathogen was CMV. The presenting infection often involves the upper and lower respiratory tract, followed by the intestinal tract, skin, urinary tract, eyes, and joints. Both of our cases also experienced repeated respiratory infections with bronchiectasis.

In addition, diarrhea has been reported in almost 50% of patients with GS [13]. Chronic diarrhea can be the sole clinical manifestation of GS at presentation [14]. The most possible cause of diarrhea associated with GS has been proposed as pathogenic infection, including *Salmonella* infection, giardiasis, CMV (duodenoenteritis), *Clostridium difficile*, and bacterial overgrowth [15]. However, some cases may also present as autoimmune enteropathy and graft-versus-host disease types of colitis. Although, for our cases, no definite pathogens were identified through the limited culture and measurement methods, the infection cannot be excluded totally. Conversely, the chronic inflammatory change and atrophy of the mucosa (seen in biopsy) in our two cases may have resulted from some unclarified pathogen(s) repeat stimulation or may be caused by some kind of immunological change, as some CVID does, resembling autoimmune enteropathy [16]. Hughes et al. [17] reported on six patients with primary hypogammaglobulinemia, among which four had malabsorption and partial-to-complete villus atrophy. These lines of evidence collectively suggest that diarrhea in hypogammaglobulinemic patients may be related to malabsorption, which is caused by intestinal mucosal lesions resembling villous atrophy.

Endoscopic and histological findings are rarely mentioned in many case reports of GS. A case reported by Mancuso et al. [14] showed that the endoscopic appearance was mild pancolitis and that the histology was suggestive of Chron’s disease. Another case reported by Verne et al. [18] showed edematous and thickened loops of bowel but a normal terminal ileum, using an upper gastrointestinal series and small bowel follow-through. Gastroduodenoscopy with small bowel biopsy in that case showed villous atrophy, with acute and chronic inflammation. To our knowledge, we were the first to evaluate the small bowel of GS patients by single-balloon enteroscopy or capsule endoscopy and present the pathologic features. Both of the procedures, for our cases, revealed mucosal edema and fine-grained or nodular appearance changes in the proximal small intestine. Histological examination showed chronic inflammation and villous atrophy. Although the two cases are characterized by repeated infection and diarrhea, the inflammatory cell infiltration was different. One case with a significant reduction on the number of peripheral blood B lymphocytes has predominant T-cell infiltration but no plasma cells in the lamina propria. While the other case with less significant reduction on the number of peripheral blood lymphocytes has predominantly CD138^+^ plasma cells but scattered CD3^+^ T-cell infiltration in the lamina propria. This was a very interesting finding. It was different from CVID that always showed lymphoid hyperplasia and no plasma cells in the lamina propria. This suggests individual variations in the nature or severity of this immunodeficiency. Thus, further studies are needed to elucidate the complex immune abnormalities that are present in GS patients, which may involve both abnormal humoral immunity and cellular immunity.

The rarity of GS has resulted in a lack of recognition. Only 6–11% of patients with thymomas have hypogammaglobulinemia, while approximately 10% of patients with adult-onset hypogammaglobulinemia may have thymoma [10,19]. The symptoms of hypogammaglobulinemia, such as infection or diarrhea, may precede or occur after the diagnosis of a thymoma, in an interval of 3 months to 23 years [11]. A systemic review of 152 cases of GS in the literature showed that the diagnosis of thymoma and the diagnosis of infection or diarrhea were not made at the same time in 42% of the patients [20], demonstrating the difficulty of GS diagnosis due to its variability in clinical manifestations. Most patients may be misdiagnosed initially as both entities infrequently present simultaneously. The two patients in our report suffered from recurrent diarrhea for 1 year and 2 years, respectively, before the diagnosis of thymoma. Although both cases were diagnosed with recurrent diarrhea, infection, and thymoma, the association between them and the possibility of GS was still not recognized. Both were misdiagnosed for 2 and 4 years, respectively, even after they had been diagnosed with thymoma. Unexplained diarrhea and recurrent infections with a history of thymoma should be clinical cues to the diagnosis of GS. Serum immunoglobulin levels, numbers of mature B-cells and chest CT findings should be considered in patients with unexplained recurrent diarrhea and opportunistic infection to diagnose GS at an early stage.

GS remains a rare entity, often associated with poor prognosis. One study reported that 33% of patients with GS were alive at 10 years after the diagnosis [20]; thymectomy, immunoglobulin replacement therapy, and anti-infectives could be the main therapies. Surgical intervention should be considered as the first-line treatment. Complete resection of thymoma [21,22] is the leading factor for long-term prognosis. Postoperatively, IVIG therapy is suggested, and it has been estimated that GS accounts for the majority among the 1–2% of primary antibody deficiency cases treated with immunoglobulin [8]. Besides, plasmapheresis and splenectomy have also been reported, with modest outcomes in regard to immune function recovery [23]. Jagessar et al. reported that fecal microbiota transplantation (FMT) has a positive influence on the treatment of refractory infections such as those in patients with GS [24].

In summary, GS is a rare cause of combined B-cell and T-cell immunodeficiency in adults with susceptibility to bacterial, viral, and fungal infections. We report herein two cases of GS with chronic recurrent diarrhea, which could be the main clinical manifestation. It was found that although GS cases shared various clinical characteristics with CVID cases, it still differed from CVID in terms of its very late onset, lack of familial cases, low or absent peripheral blood B lymphocytes, absence of lymphoid hyperplasia, and plasma cell infiltration in the lamina propria in some patients. These differences between them suggested that GS might be an acquired immunodeficiency, part of an increasingly recognized group of adult-onset immunodeficiency disorders associated with thymoma. Awareness of thymoma accompanied by hypogammaglobulinemia as a cause of diarrhea can help in reaching the correct diagnosis. Further studies are needed to explain why some patients with thymoma develop persistent hypogammaglobulinemia and individual variations in the nature or severity of this immunodeficiency.

## Abbreviations

CMV: cytomegalovirus
CT: computed tomography
CVID: common variable immunodeficiency
EBV: Epstein-Barr virus
FMT: fecal microbiota transplantations
GS: Good’s syndrome
HIV: human immunodeficiency virus
HSV: herpes simplex virus
IVIG: intravenous immunoglobulin
UKPID: UK-primary immune deficiency
